# Pattern recognition receptors in antifungal immunity

**DOI:** 10.1007/s00281-014-0462-4

**Published:** 2014-11-25

**Authors:** Anthony Plato, Sarah E. Hardison, Gordon D. Brown

**Affiliations:** Division of Applied Medicine Immunity, Infection and Inflammation Programme Room 4.20, Institute of Medical Sciences, Ashgrove Road West University of Aberdeen, Aberdeen, AB25 2ZD UK

**Keywords:** C-type Lectin, Dectin-1, Syk, Antifungal, Th17

## Abstract

Receptors of the innate immune system are the first line of defence against infection, being able to recognise and initiate an inflammatory response to invading microorganisms. The Toll-like (TLR), NOD-like (NLR), RIG-I-like (RLR) and C-type lectin-like receptors (CLR) are four receptor families that contribute to the recognition of a vast range of species, including fungi. Many of these pattern recognition receptors (PRRs) are able to initiate innate immunity and polarise adaptive responses upon the recognition of fungal cell wall components and other conserved molecular patterns, including fungal nucleic acids. These receptors induce effective mechanisms of fungal clearance in normal hosts, but medical interventions, immunosuppression or genetic predisposition can lead to susceptibility to fungal infections. In this review, we highlight the importance of PRRs in fungal infection, specifically CLRs, which are the major PRR involved. We will describe specific PRRs in detail, the importance of receptor collaboration in fungal recognition and clearance, and describe how genetic aberrations in PRRs can contribute to disease pathology.

Fungi are usually efficiently cleared by an intact immune system; however, during immunosuppression, susceptibility to fungi increases and infection can be associated with high mortality rates [1]. Advances in medical technologies such as chemotherapy or indwelling medical devices and the appearance of immunosuppressive viruses such as HIV have led to a marked increase in the incidence of invasive fungal infection in the last several decades. Superficial infections in immunocompetent patients are also prevalent, including fungal keratitis and chronic skin diseases such as chromoblastomycosis [2, 3]. Coupled with the lack of reliable and swift identification methods and limited treatment options, incidence and morbidity due to fungal infections is currently at an unacceptably high rate.

Pattern recognition receptors (PRR) are germline-encoded receptors that recognise a variety of pathogen-associated molecules (pathogen-associated molecular patterns, PAMPs) expressed by an invading microorganism. These receptors induce downstream events designed to eliminate the pathogen from the host, including phagocytosis, respiratory burst, and cytokine and chemokine release. PRRs are best characterised into one of four families: the Toll-like (TLR), NOD-like (NLR), RIG-I-like (RLR) and C-type lectin-like receptors (CLR), each of which differ in ligand recognition, signal transduction and sub-cellular localisation. Most PRRs are expressed on dendritic cells (DCs) and other myeloid cells and are notable for initiating innate immune defences; however, PRR signalling can also direct the development of the adaptive immune response by secreting cytokines which polarise CD4^+^ T cells (T-helper or Th cells) [4, 5]. For fungi, Th1 and/or Th17 immunity is crucial for clearance of infection [6, 7].

Our current knowledge demonstrates that CLRs are the major receptor group for the recognition of fungi, while TLRs and NLRs play ancillary roles. This review will assess the current knowledge of selected PRRs in the pathogenesis of fungal infection, in particular how defects in host PRR signalling and immune evasion tactics allow fungal pathogens to thrive in the host.

## C-type lectin-like receptors

CLRs are transmembrane receptors expressed on, but not restricted to, myeloid cells including macrophages and DCs. CLRs can also be found on lymphocytes, granulocytes, osteoclasts and epithelial cells [8–11]. CLRs are able to recognise a diverse range of ligands via one or more C-type lectin-like domains (CTLD). Evolutionary diversion of this domain from the carbohydrate recognition domain (CRD) of classical C-type lectins has allowed them to recognise a range of molecules including carbohydrates, lipids and proteins [11, 12]. CLRs recognise both endogenous and exogenous ligands in the regulation of homeostasis and immunity, utilising spleen tyrosine kinase (syk) as a primary signal transduction molecule. Signalling commonly requires an immunoreceptor tyrosine-based activation motif (ITAM) or ITAM-like motif which is phosphorylated at tyrosine(s) within the consensus sequences (YxxI/L _(6–12)_YxxI/L) and (YxxI/L) respectively. This motif can be present within the signalling tail of the receptor itself or an associated adapter chain such as FcRγ. As well as cellular activation, many CLRs can perform inhibitory roles by utilising an immunoreceptor tyrosine-based inhibitory motif (ITIM). Phosphorylation of the central tyrosine within the consensus sequence (I/V/L/SxYxxI/L/V) is involved in the recruitment of tyrosine phosphatases, exhibiting regulatory effects on other activation pathways [13].

**Fig. 1:**
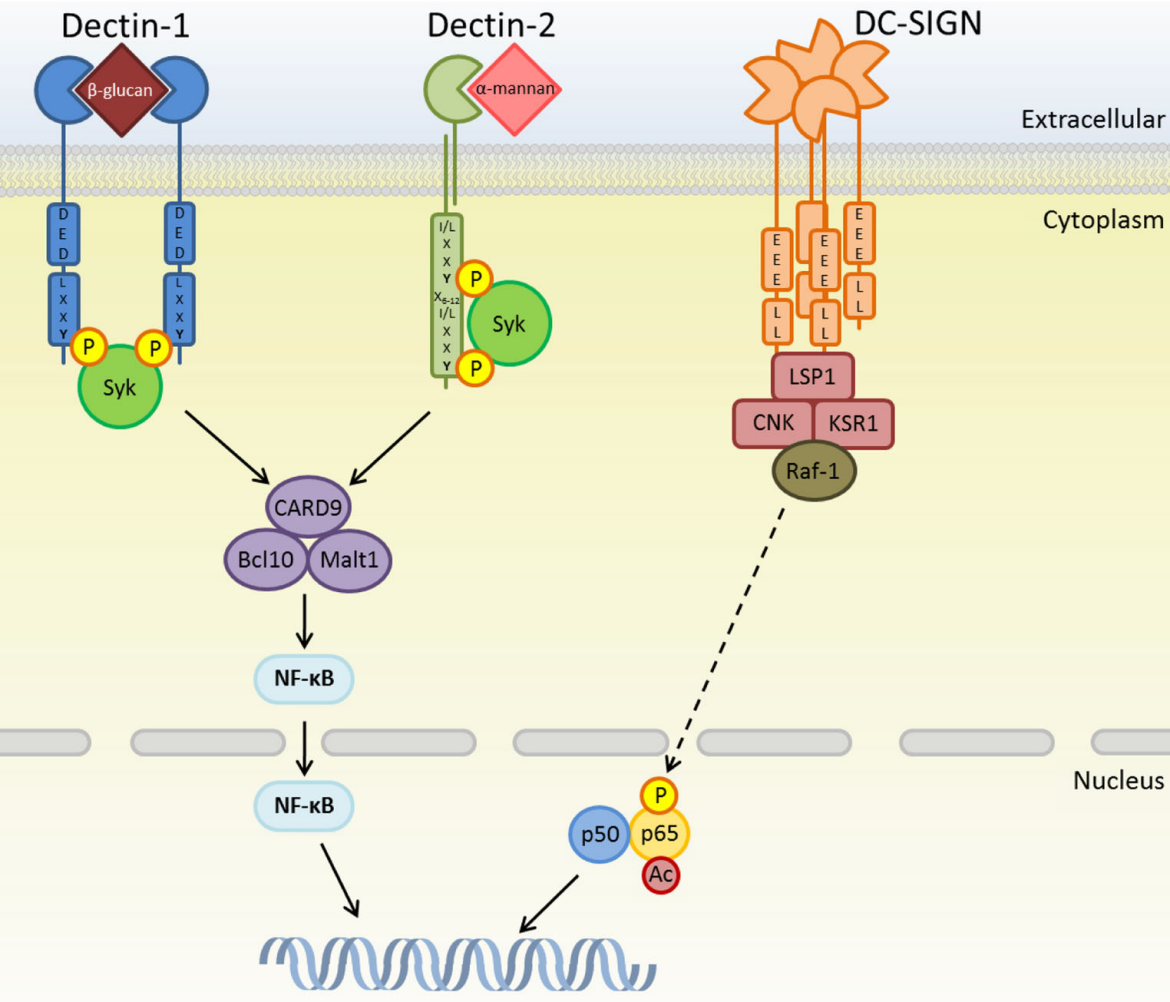
Examples of activation signals initiated by CLRs: both Dectin-1 and Dectin-2 signal via tyrosine-based motifs (ITAM-like and FcRγ-associated ITAM, respectively) where recruitment of syk directly to phosphorylated tyrosine residues results in downstream activation of the CARD9-Bcl10-Malt1 complex. DC-SIGN contains no tyrosine-based motifs but is able to induce phosphorylation and acetylation of NF-κB subunit p65 via the assembly of signalosome. DC-SIGN activation of p65 is Raf-1-dependent

Current understanding is that ITAM motifs are phosphorylated by Src family kinase members, allowing the recruitment and subsequent activation of syk. A large network of proteins including phospholipase Cγ2 (PLCγ2), protein kinase Cδ (PKCδ), IκB kinase (IKK) and the CARD9-Bcl10-MALT1 complex is known to exist downstream of syk and upon activation, these molecules regulate effects specific to each receptor [14]. The CLRs Dectin-1, Dectin-2, CLEC-9A, CLEC-2, and macrophage-inducible C-type lectin (Mincle) signal via syk, but their biological roles can differ greatly. Currently, CLEC-9A and CLEC-2 are known to have important roles in cell damage detection and platelet activation, respectively, whereas Dectin-1, Dectin-2 and Mincle play major roles fungal and bacterial infections [14, 15]. On the other hand, ligand binding to ITIM-containing receptors results in ITIM phosphorylation and the association of SH2-domain-containing protein tyrosine phosphatases including (SHP)-1 and SHP-2, that are able to directly regulate signalling downstream of ITAM motifs. CLRs such as DCIR, MICL and MAH contain ITIM motifs [14]; however, in this review, we will focus on the activation of CLRs related to fungal disease (Fig. 1).

## Dectin-1/CARD9

Dectin-1 is a well-defined CLR that recognises β-1,3-glucans present in the cell wall of *Aspergillus*, *Candida* and many other fungal species [8, 16]. Dectin-1 contains a single CTLD that is able to recognise fungal β-glucans and undefined ligands on mycobacteria and T cells, although vimentin was recently identified as possible endogenous Dectin-1 ligand [17]. Upon ligand recognition, Dectin-1 signals through the syk/CARD9 pathway via an ITAM-like motif. This motif is present in the intracellular signalling tail of Dectin-1 and uses a single phosphorylatable tyrosine residue to recruit syk to dimerised Dectin-1 [18]. Through phosphorylation of phospholipase C (PLC)γ2 and subsequent activation of protein kinase C (PKC), syk activates the CARD9-Bcl10-MALT1 complex to induce canonical NF-κB activation. Syk can also induce non-canonical NF-κB activation through NK-κB-inducing kinase (NIK) [19–21]. These pathways induce cytokine and chemokine production including TNF, IL-2, IL-10, CXCL2 as well as IL-1β, IL-6 and IL-23, key cytokines in the development of the antifungal Th17 response, as discussed later [22–24]. Dectin-1 also induces phagocytosis, respiratory burst and other antimicrobial effector mechanisms such as activation of the NLRP3 inflammasome via ERK-induced reactive oxygen species (ROS) production [14, 25]. The role of the inflammasome in antifungal immunity will be discussed in more detail later [26].

The importance of Dectin-1 in the control of fungal infections is highlighted by the susceptibility of Dectin-1-deficient mice to infection with *Candida albicans*, *Aspergillus fumigatus* and *Pneumocystis carinii* [22, 27, 28]. Interestingly, the degree of susceptibility is strain-dependent in the case of *C. albicans* [29]. Furthermore, Dectin-1 deficiency in humans can lead to susceptibility to certain fungal infections including chronic mucocutaneous candidiasis and recurrent vulvovaginal candidiasis [30, 31]. Both of these infections occur more frequently in patients containing a mutation in Dectin-1 (Y238X) which results in the production of a truncated protein. This truncation results in a loss of function and as such, monocytes and macrophages from patients with this polymorphism are deficient in cytokine secretion following stimulation with particulate β-glucan or *C. albicans* [30]. The Y238X polymorphism has also been shown in some studies to be associated with susceptibility to invasive aspergillosis with defective IFNγ, IL-10, IL-1β, IL-6 and IL-17 responses [32]. Recently, a polymorphism in Dectin-1 was identified that is associated with severe, intractable forms of ulcerative colitis resulting from improper recognition of fungal gut microbes [33].

Predisposition to fungal infections can also be seen in CARD9-deficient humans. CARD9 deficiency has been studied in a family in which eight members were affected with a mutation causing a premature stop codon (Q295X), resulting in a truncated protein [31]. Three of these individuals died from invasive candida infections of the brain in early childhood, while four family members were affected by recurrent mucocutaneous candidiasis.

## Dectin-2/Mincle

The roles of Dectin-2 and Mincle in immunity are well-characterised, and both recognise fungi. Unlike Dectin-1, the CTLD of Dectin-2 is more like a classical CRD, containing the short Glu-Pro-Asn (or EPN) motif that recognises mannose structures in a calcium-dependent manner. For both Dectin-2 and Mincle, surface expression and signal transduction require an associated FcRγ signalling adapter that contains an ITAM motif. The two tyrosines within the ITAM consensus sequence are phosphorylated by Src family kinases, allowing recruitment of syk to the activated receptor and signalling via the CARD9-Bcl10-MALT1 complex. Cytokine transcription is initiated via ERK, p38 and MAP kinases [15, 34].

Dectin-2 has been shown to recognise *C. albicans*, *A. fumigatus*, *Saccharomyces cerevisiae*, *Cryptococcus neoformans*, *Histoplasma capsulatum*, *Trichophyton rubrum*, *Microsporum audouinii* and *Malassezia* spp. Dectin-2^−/−^ bone marrow-derived dendritic cells (BMDC) are unable to induce cytokine responses to *C. albicans*, including IL-1β, IL-6, TNF, IL-12p40 and IL-10, and Dectin-2^−/−^ mice are more susceptible to *C. albicans* infection. Furthermore, Dectin-2^−/−^ mice are deficient in Th17 polarisation in response to *C. albicans*, an effect dependent on the syk/CARD9 pathway [35]. Activation of Dectin-2 on human DCs selectively activates the NF-κB subunit c-Rel via Malt1 activation. This is unlike Dectin-1, where ligand recognition results in activation of all NF-κB subunits [21, 34]. MALT1/c-Rel activation by Dectin-1 and Dectin-2 was shown to be important for the induction of a Th17 response [34–36]. Susceptibility to fungal disease due to Dectin-2 polymorphisms has not yet been described in humans.

While Mincle is primarily known for its role in recognising mycobacteria, this CLR is also able to recognise fungal species including *Candida*, *Malassezia* and Fonsecaea species [1, 37]. Recognition of these fungi by Mincle can lead to phagocytosis, fungal killing and induction of TNF, IL-6, MIP-2 and KC in macrophages [15, 38, 39], and clearance of murine *Fonsesaea pedrosoi* infection is dependent on Mincle [40]. The role of Mincle in anti-Candida immunity is controversial, with contrasting data on the importance of this receptor in vivo [38, 41]. Similarly to Dectin-2, there are no known human polymorphisms in Mincle that lead to susceptibility to fungi.

## DC-SIGN

Dendritic cell-specific intercellular adhesion molecule-3 grabbing non-integrin (DC-SIGN, murine homolog SIGNR1) is a transmembrane CLR that contains a single CRD that recognises carbohydrate-based ligands including mannose and fucose structures. Unlike the other C-type lectins already discussed, DC-SIGN does not have tyrosine-based signalling motifs within its intracellular tail, instead it contains di-leucine (LL) and tri-acidic (EEE) motifs. Very little is known about the signal transduction pathways utilised by DC-SIGN; however, it has been shown that DC-SIGN can activate Raf-1, a serine/threonine MAP kinase that is involved in the acetylation the NF-κB subunit, p65. Interestingly, this acetylation only takes place on p65 molecules that were previously activated by TLRs. This acetylation enhances and prolongs transcription of the IL-10 gene, leading to an enhanced anti-inflammatory response [42]. Interactions of CLRs and TLRs such as this are crucial to antifungal immunity, and will be discussed in greater detail in following sections [14, 24, 42, 43].

**Fig. 2:**
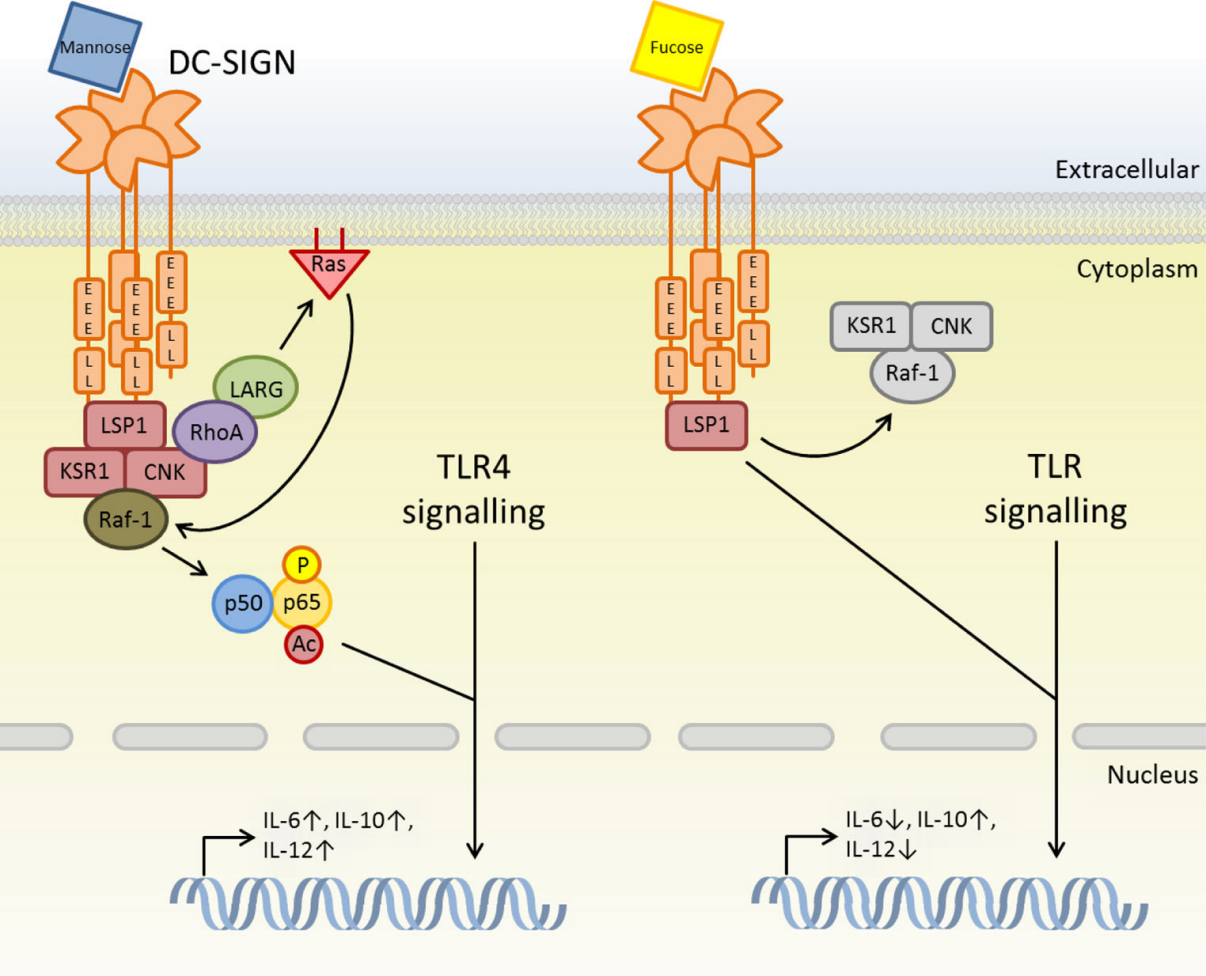
DC-SIGN pathogen recognition determines downstream TLR regulation. Formation or disassembly of the DC-SIGN signalosome is able to tailor TLR signalling pathways towards a more effective response to each pathogen encountered. This is a novel mechanism for pathogen differentiation including potential fungal mannan-induced signalling

The ability of DC-SIGN to recognise varied carbohydrate structures allows it to differentiate between diverse organisms such as *C. albicans*, *Mycobacterium tuberculosis*, HIV-1, or *Helicobacter pylori*, allowing a tailored response to each through the formation of the DC-SIGN signalosome (Fig. 2). The DC-SIGN signalosome is a complex required for the constitutive association of DC-SIGN with Raf-1. This complex is made up of the scaffold proteins LSP1, KSR1 and CNK, and the association or dissociation of this complex allows varying inflammatory responses depending on the ligand associated. For example, IL-10, IL-12 and IL-6 expression is observed upon mannose recognition. Here, recruitment of LARG and RhoA to the signalosome results in activation of Ras and Raf-1 for the regulation of TLR4 pathways. On the other hand, fucose stimulation of DC-SIGN results in the dissociation of this complex and the down-regulation of IL-6 and IL-12 in an LSP-dependent manner. [43]. Interestingly, DC-SIGN can also cooperate with the mannose receptor (MR) to repress Dectin-1-dependent Th17 development in human DCs upon stimulation with either β-glucans or *M. tuberculosis* [44].

DC-SIGN is also able to recognise *A. fumigatus*, and human polymorphisms in DC-SIGN cause increased susceptibility to invasive pulmonary aspergillosis (IPA), a life-threatening infection of respiratory tracts often seen in patients with neutropenia or other immunosuppression [45, 46]. Several single nucleotide polymorphisms (SNPs) were shown to increase the risk of developing IPA by 2-fold in patients that had undergone haematopoietic stem cell transplantation (HSCT) or chemotherapy.

## Mannose receptor

The MR is a CLR that recognises multiple fungi, bacteria and viruses via eight extracellular CTLDs. Specifically, the CTLD recognises terminal mannose and fucose-based structures as well as N-acetyl glucosamine [24]. Unlike the other CLRs discussed here, the MR extracellular domain can be cleaved by a metalloproteinase to form soluble MR (sMR). MR shedding was shown to be induced by Dectin-1 recognition of zymosan, *C. albicans*, *A. fumigatus* and cell wall components of *S. cerevisiae*. Interestingly, sMR is still able to recognise pathogen and host molecules [47]. The MR is able to induce ROS and cytokine production; however, as its signalling tail lacks known motifs; it is thought that responses to *C. albicans* involve collaboration with Dectin-1 and peroxisome proliferator activated receptor γ (PPARγ) [48]. MR is known to play a role in the development of Th17 responses to *C. albicans*; however, MR knockouts are not susceptible to *C. albicans* infection [49].

## TOLL-like receptors

Although CLRs are considered to be the primary receptor class for fungal recognition, members of the TLR family and their interactions with CLRs are critical to antifungal immunity. TLRs are considered to be the most well-defined of the four families of PRRs. There are 13 members in mice, and TLR1-10 are expressed in humans. All of these members are transmembrane receptors expressed either at the cell surface, in the case of TLR1, 2, 4, 5, 6 and 10, or on intracellular membranes in the case of TLR3, 7, 8, 9. Transmembrane TLRs recognise PAMPs present in cell walls and membranes, for example, lipopolysaccharide (LPS) and peptidoglycan, which are recognised by TLR4 and TLR2, respectively. Alternatively, TLRs present on intracellular membranes recognise fungal, bacterial and viral nucleic acids within an infected cell. TLRs (with the exception of TLR3) are all able to signal through MyD88, which has been shown to be crucial in the clearance of infections. MyD88^−/−^ mice have been shown to be sensitive to fungal infections, including *C. albicans* and *A. fumigatus*, and MyD88 is required for optimal Th1 responses to fungi in mice [50].

Individually, TLR2, 4, 7 and 9 have been shown to play roles in *C. albicans* infections in mice. TLR4-defective (C3H/HeJ) mice are more susceptible to disseminated candidiasis, characterised by increased kidney fungal burdens and reduced neutrophil recruitment [51]. Similarly, TLR2^−/−^ mice are susceptible to systemic challenge with *C. albicans*. [52]. It should be noted, however, that TLR recognition of different *C. albicans* strains as well as other fungi is highly variable, leading to some confusion in the literature [53]

In humans, complete lack of TLR functionality, through mutation of key downstream signalling adaptors such as MyD88 does not predispose individuals to fungal infections [54]. This demonstrates that TLRs are not the central PRRs involved in antifungal immunity in humans. However, in immunocompromised hosts, polymorphisms in the TLR genes can result in a predisposition to fungal infections [55–58]. For example, D299G polymorphism in TLR4 increases susceptibility to chronic cavity pulmonary aspergillosis (CCPA), a subacute cavity-forming aspergillosis. Interestingly, TLR2 SNPs showed no susceptibility to CCPA but a potentially protective role in allergic bronchopulmonary aspergillosis (ABPA), whereas patients suffering from ABPA had significantly higher frequency of a TLR9 SNP (T-1237C) compared to unaffected controls [58].

## Collaboration between CLRs and TLRs

**Fig. 3:**
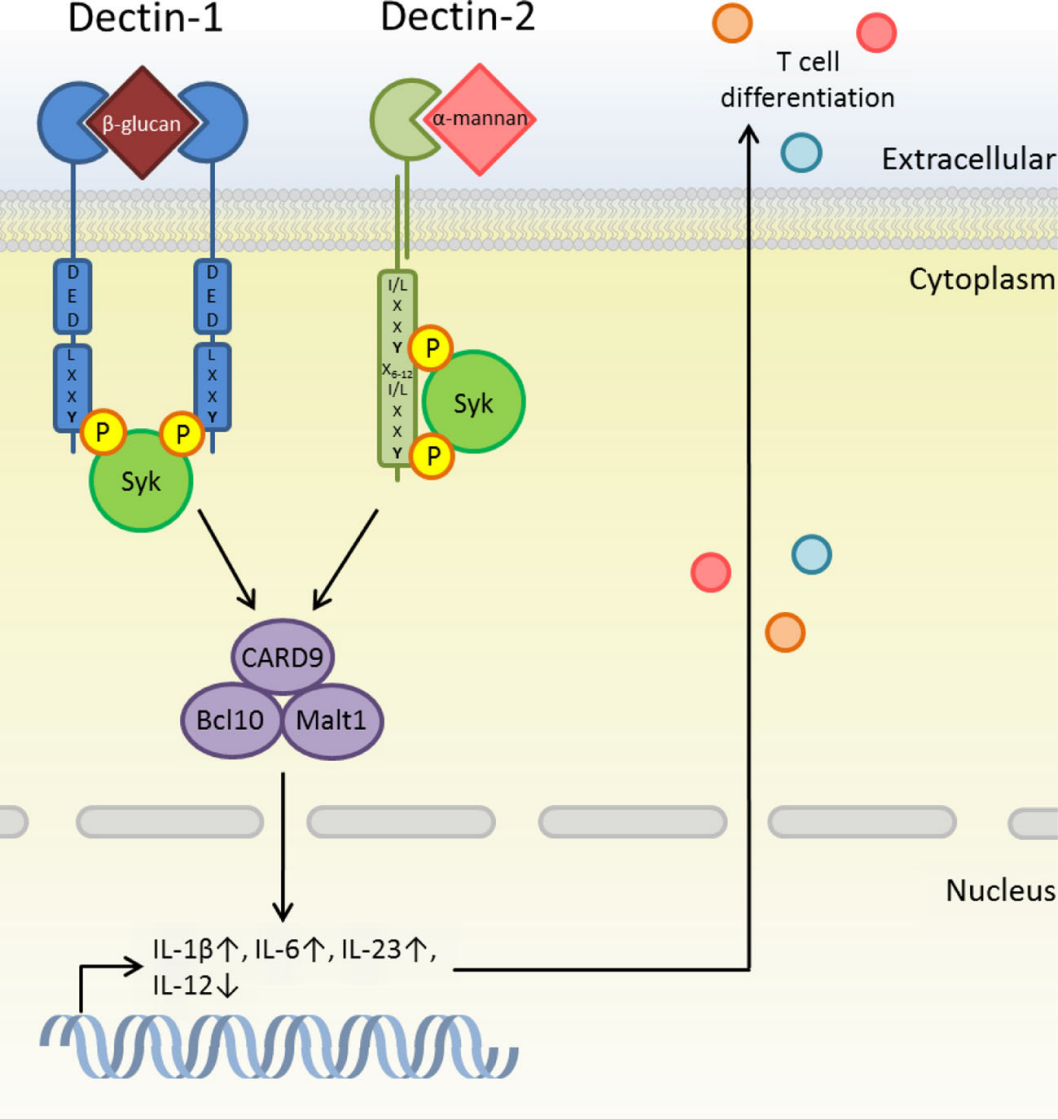
Dectin-1 and Dectin-2 contribute to T cell differentiation: Dectin-1 and Dectin-2 are able to induce Th17-polarising cytokine secretion from DCs upon ligand activation and subsequent syk/CARD9 activation. As well as this IL-17 production can be directly induced by Dectin-1 activation from γδ T cells, increasing anti-fungal activity

TLRs and CLRs are both required for antifungal responses, and these pathways interact to induce optimal immunity dependent on Syk/CARD9 and MyD88 signalling pathways [1, 59, 60]. For example, co-stimulation with both Dectin-1 and TLR2 or TLR4 ligands leads to a synergistic increase in TNF, IL-10 and IL-23 and a decrease in IL-12 production (Fig. 3) [1, 60, 61]. SIGNR1, a murine homolog of DC-SIGN, also collaborates with TLRs in the recognition of *C. albicans*. Recent studies have demonstrated that SIGNR1 suppresses the function of TLR2 by down-regulating TNF, IL-6, IL-12p40 and FSL-1-mediated activation of NF-κB [62]. However, in collaboration with TLR2 and Dectin-1, SIGNR1-expressing macrophages were shown to induce TNF production [63]. These functions are not abolished by mutation of the SIGN-R1 intracellular tail, suggesting no signalling role for SIGN-R1; however, the overall mechanism is still unclear [63, 64]. It is hypothesised that this receptor aids binding of fungi at the cell surface, or that it interacts with other receptors at the site of microbe contact.

Modulating CLR and TLR collaboration has been utilised therapeutically in the treatment of fungal infections, such as for the clearance of *F. pedrosoi* from infected mice. *F. pedrosoi* is the causative agent of the chronic skin disease chromoblastomycosis, and is recognised by Mincle. Patients with severe *F. pedrosoi* infection may have prolonged antifungal treatment courses [65]; however, recent studies have shown that application of topical TLR7 ligand, imiquimod, results in rapid clearance of infection in mice as well as small cohort of human patients [1, 37, 40]. Similar observations of CLR and TLR collaboration were made for the reduction of *P. carinii* lung burden in mice. Treatment with aerosolised heat-killed *E. coli* was able to induce clearance of the infection, likely through Dectin-1 and TLR2 collaboration [66, 67].

Recently, collaboration between different CLRs has also been described. For example, Mincle and Macrophage C-type lectin (MCL, also CLECSF8 or CLEC4D) form a receptor complex with the signalling adaptor FcRγ to induce downstream signalling through syk/CARD9. Both receptors and FcRγ were shown to be required for optimal phagocytosis [68, 69]. Furthermore, Mincle activation was recently shown to inhibit Dectin-1 induced proinflammatory responses [70]. Further investigation of multiple CLR interactions may answer questions about other instances of modified ligand recognition during receptor complex formation.

## NOD-like receptors

NLRs are cytoplasmic receptors that recognise PAMPs of internalised microbial components. Currently, there are 23 known human NOD-like receptors that are broadly categorised into two subfamilies: the NOD subfamily, characterised by the presence of one or more CARD domain, and the NOD leucine-rich repeat and pyrin domain-containing protein (NLRP) subfamily characterised by the presence of a pyrin domain. Notable members of the NOD subfamily include NOD1 and NOD2. These receptors are well studied, in particular the role of NOD2 in Crohn’s disease; however, the receptors of importance in antifungal immunity are those that form an inflammasome complex. Inflammasomes are innate immune complexes that cleave pro-IL-1β and pro-IL-18 into their biologically active forms. Cleavage of pro-IL-1β/18 is often considered the secondary signal to IL-1β/18 production, the first signal being PAMP recognition and production of pro-IL-1β/18 by NF-κB activation [26, 71]. NLRs capable for forming inflammasomes include NLRP1, 2, 3, 6, 7 and NLRC4 and 5 where, upon ligand activation, they associate with ASC and caspase-1 to form the basis of the inflammasome [72].

Although its ligand is yet to be defined, NLRP3 is a well-studied inflammasome NLR and is known to contribute to antifungal immunity [26, 71, 73]. Observations of NLRP3 revealed that it is essential in the control of mucosal and disseminated *C. albicans* infections by the processing of pro-IL-1β to IL-1β downstream of Dectin-1 and TLR2 [71]. Importantly, NLRP3 recognition of *C. albicans* requires the fungus to be in a filamentous state, as *C. albicans* incapable of forming hyphae do not induce this response [74]. Both caspase-1^−/−^ and ASC^−/−^ mice were susceptible to *C. albicans* infection compared to wild-type mice, likely due to increased fungal burdens in both knockouts as well as tissue invasion in the caspase-1^−/−^ mice. Reduced IL-1 and IL-17 were found at the site of infection as well as little neutrophil infiltration. BMDCs from these knockouts also showed defective production of IL-1β and IL-18, cytokines responsible for Th17 and Th1 polarisation, respectively, suggesting that the NLRP3 inflammasome can control T-helper responses to *C. albicans* infection [75]. NLRP3 has also been shown to induce IL-1β maturation in response to *A. fumigatus* hyphae and relies upon Dectin-1 activation of Syk as well as ROS production and K + efflux [73]. Furthermore, using bone marrow chimera studies, NLRC4 has been demonstrated to be important for controlling candida infection at mucosal sites [76].

Interestingly, the caspase-8 inflammasome is able to induce IL-1β independently of NLRs in a manner directly through Dectin-1 signalling. In contrast to the NLRP3 inflammasome, caspase-8 and ASC are recruited directly to the CARD9-Bcl10-MALT1 signalling complex, where IL-1β cleavage takes place. This pathway also activates the non-canonical NF-κB subunit c-Rel which induces transcription of the IL1β gene, allowing an effective antifungal response through rapid IL-1β production [77].

## The TH17 response in antifungal immunity

Th17 cells are a subset of CD4^+^ T cells involved in protective immunity to fungal infections, and these responses appear to be primarily triggered through CLRs. Defects in Th17 defence often result in recurring fungal infection and autoimmune disease [24, 78, 79]. Differentiation and development of Th17 cells requires TGF-β, IL-6 and IL-21 along with IL-23 for growth and increased Th17 response. Transcription factors including signal transducer and activator of transcription 3 (STAT3), RAR-related orphan receptor (ROR)α and RORγt are also required for Th17 development and maintenance [80, 81]. Th17 cells produce the cytokines IL-17A and F that bind the IL-17 receptor and induce the secretion of proinflammatory factors including IL-6, IL-8 and GM-CSF as well as the chemokines CXCL1 and CCL20, which are involved in macrophage and neutrophil recruitment [82]. This strong proinflammatory effect can be seen in stromal cells of almost all tissues [83].

CLRs are critical to the development of Th17 cells during infection. In particular, Dectin-1 is able to regulate differentiation of CD4^+^ T cells into Th17 cells by inducing DC activation through recognition of β-glucan and cytokine production. This is dependent on both syk/CARD9 and Raf-1 pathways where Th17-promoting cytokines IL-1β, IL-6 and IL-23 are increased and Th1-promoting cytokine IL-12 is decreased [60, 84]. Dectin-2 shows a similar cytokine profile upon recognition of *C. albicans*, and when blocked, IL-17 production from T cells was prevented, suggesting Dectin-2 plays a pivotal role in *Candida*-specific Th17 responses [34, 35]. Dectin-1-expressing T cell receptor gamma-delta (TCRγδ) T cells have also been shown to induce production of IL-17 upon stimulation with β-glucan and curdlan. These γδ T cells are thought to be important during an early innate response, as they are potent sources of IL-17. The innate response can be further amplified in the presence of IL-23, increasing IL-17 production, T cell expansion and neutrophil recruitment [85, 86]. Additionally, IL-17 can stimulate epithelial cells to produce neutrophil-recruiting chemokines and antimicrobial peptides such as β-defensin and the S100A proteins [87].

Evidence of a protective role for Th17 cells have been shown for multiple murine studies; however, it is apparent that uncontrolled Th17 inflammation is deleterious. A lack of Th17 polarisation leads to increased susceptibility to fungal infections. For example, IL-23p19^−/−^ mice, which are Th17 impaired, lack effective clearance of *P. carinii* as well as reduced CD4^+^ T cells in the lungs [88]. IL-17A receptor-deficient mice are severely impaired for clearance of *C. albicans*, and increased mortality is observed in a model of systemic candidiasis. Alternatively, driving Th17 responses during *C. albicans* infection reduces Th1 immunity and results in generalised neutrophilic inflammation which exacerbates disease [89]. Th17 differentiation in response to *C. albicans* is initiated by Dectin-1 via the syk-CARD9 complex, and is independent of TLR signalling. Recently, deficiencies in neutrophil recruitment to sites of infection have been observed in an oropharyngeal candidiasis (OPC) model in IL-17AR^−/−^ and IL-23^−/−^ mice [90]. Neutrophils exhibit IL-17 autocrine activity that induces innate responses such as ROS production and increased fungal killing. These responses are induced by IL-6 and IL-23, and were completely dependent on RORγt, suggesting the importance of neutrophils in Th17 responses [2]. Recently, IL-17 was shown to be important for controlling the ability of NK cells to produce GM-CSF, a cytokine required to promote neutrophil antifungal activities and essential for the control of systemic Candida infections [91]. In humans, polymorphisms in genes which regulate Th17 differentiation, such as IL-17, IL-17RA, STAT1 or STAT3, result in susceptibility to mucocutaneous candidiasis, demonstrating the essential nature of this pathway in antifungal defence [92–94].

### Immune evasion

Fungi can utilise differential expression of PAMPs to evade recognition by the immune system. Masking or modification of cell wall components can prevent PRR recognition. β-1,3-glucans are highly immunostimulatory and are often masked by less immunogenic structures in the outer cell wall [95]. For example, *C. albicans* is able to mask β-1,3-glucan under a layer of mannoproteins, preventing recognition by Dectin-1. Although mannoproteins themselves are immunostimulatory, it appears that the lack of β-glucan exposure results in a defective co-stimulatory response between Dectin-1 and TLR2/4 [96]. Surface β-glucan exposure is increased at bud scar; however, it is masked by mannans in the hyphal form. Growth in the hyphal form could be seen as an evasion tactic, as Dectin-1 has a reduced affinity for hyphal *C. albicans* compared to the yeast form [97, 98]. In contrast, Dectin-2 can also recognise *C. albicans*, but is thought to preferentially bind the hyphal morphology [99].

Masking of immunostimulatory molecules is observed in other species of pathogenic fungus. For example, *H. capsulatum*, the causative agent of the pulmonary disease histoplasmosis, employs α-1,3-glucans to cover the β-1,3-glucan underneath. In the presence of this α-glucan layer, TNF production is suppressed, suggesting this is an effective mechanism in reducing proinflammatory responses [100]. Alternatively, *P. brasiliensis*, a fungus known to cause the systemic infection paracoccidioidomycosis, reduces PAMP surface expression by converting the cell wall glucan linkage from β-1,3 to α-1,3-glucans during its transition into pathogenic yeast form. *C. neoformans* also evades immune surveillance by masking its PAMPs under a thick layer of polysaccharides known as the capsule. Control of the capsule’s structure allows *C. neoformans* to survive extracellular stresses [101]. Acapsular mutants of *C. neoformans* are avirulent, demonstrating the efficacy of the capsule in preventing efficient clearance from the host [102]. Interestingly, the capsule can also bind mannan-binding lectin, preventing complement activation [103, 104].

## Conclusions

PRRs are the first line of defence in antifungal immunity. Upon recognition of surface-expressed PAMPs such as β-glucan and α-mannan, CLRs initiate inflammatory innate responses that in turn polarise a Th17 adaptive immune response which is generally beneficial to fungal control and clearance. When these immune defences fail, such as in immunocompromised hosts, opportunistic fungal pathogens are able to establish potentially life-threatening infections. Our current understanding of receptor partnering is developing, and future work may shed light on how cooperation between CLRs and TLRs collectively recognises different microbes and shape the development of immunity. It is clear that host-pathogen interactions are highly complex, and understanding these mechanisms will be crucial in developing effective treatments against fungal infections in the future.
